# Successive Passage In Vitro Led to Lower Virulence and Higher Titer of A Variant Porcine Epidemic Diarrhea Virus

**DOI:** 10.3390/v12040391

**Published:** 2020-04-01

**Authors:** Pengwei Zhao, Song Wang, Zhi Chen, Jiang Yu, Rongzhi Tang, Wenbin Qiu, Lu Zhao, Yueyue Liu, Xiaozhen Guo, Hongbin He, Guanlong Xu, Jinxiang Li, Jiaqiang Wu

**Affiliations:** 1 Department of Biochemistry and Molecular Biology, School of Medicine, Zhejiang University, Hangzhou 310058, China; 2 Ruminant Diseases Research Center, Key Laboratory of Animal Resistant Biology of Shandong, College of Life Sciences, Shandong Normal University, Jinan 250014, China; 3 Shandong Provincial Key Laboratory of Animal Diseases Control and Breeding, Shandong Academy of Agricultural Sciences, Jinan 250100, China; 4 Shandong Key Laboratory of Poultry Diseases Diagnosis and Immunology, Shandong Academy of Agricultural Sciences, Jinan 250023, China; 5 China Institute of Veterinary Drug Control, Beijing 100081, China; 6 National Agricultural Science and Technology Center, Chengdu 610000, China

**Keywords:** porcine epidemic diarrhea virus, virus isolate, variant, pathogenicity, cytokines

## Abstract

A highly virulent porcine epidemic diarrhea virus (PEDV) appeared in China and spread rapidly to neighbor countries, which have led to great economic losses to the pig industry. In the present study, we isolated a PEDV using Vero cells and serially propagated 100 passages. PEDV SDSX16 was characterized in vitro and in vivo. The viral titers increased to 10^7.6^ TCID_50_/mL (100th) by serial passages. The spike (S) gene and the whole gene of the SDSX16 virus was fully sequenced to assess the genetic stability and relatedness to previously identified PEDV. Along with successive passage in vitro, there were 18 nucleotides (nt) deletion occurred in the spike (S) gene resulting in a deletion of six amino acids when the SDSX16 strain was passaged to the 64th generation, and this deletion was stable until the P100. However, the ORF1a/b, M, N, E, and ORF3 genes had only a few point mutations in amino acids and no deletions. According to growth kinetics experiments, the SDSX16 deletion strain significantly enhanced its replication in Vero cells since it was passaged to the 64th generation. The animal studies showed that PEDV SDSX16-P10 caused more severe diarrhea and vomiting, fecal shedding, and acute atrophic enteritis than SDSX16-P75, indicating that SDSX16-P10 is enteropathogenic in the natural host, and the pathogenicity of SDSX16 decreased with successive passage in vitro. However, SDSX16-P10 was found to cause lower levels of cytokine expression than SDSX16-P75 using real-time PCR and flow cytometry, such as IL1β, IL6, IFN-β, TNF-α, indicating that SDSX16-P10 might inhibit the expression of cytokines. Our data indicated that successive passage in vitro resulted in virulent attenuation in vivo of the PEDV variant strain SDSX16.

## 1. Introduction

Porcine epidemic diarrhea (PED) is a highly contagious swine disease. The main clinical signs include severe vomiting, diarrhea, and dehydration in suckling piglets [1,2,3]. The disease was first identified in the United Kingdom in 1971, and outbreaks have been reported in many pig-producing countries in Europe and Asia [4,5,6,7]. PED outbreaks have recently appeared in the United States [8] and rapidly spread in North America, including Mexico and Canada [9], causing significant economic losses to the swine industry [10,11]. PED is caused by porcine epidemic diarrhea virus (PEDV), and belongs to the genus *Alphacoronavirus* in the sub-family *Coronavirinae* [12]. The PEDV has a 28-kb, single-stranded, positive-sense RNA genome that encodes four structural proteins, including spike (S), envelope (E), membrane (M), nucleocapsid (N) [13]. The spike (S) protein is an important envelope glycoprotein of the virus and plays a vital role in interacting with cellular receptors for virus entry, mediating cell fusion, and induction of neutralizing antibodies. Thus, the S protein is considered a suitable candidate for examining the epidemiological status of the virus in the field, strain diversity, the association between gene mutations and viral antigenicity, and developing diagnostic assays and vaccines [14,15,16,17]. Although the most of sow herds had immunized with a CV777-based vaccine, large-scale outbreaks of PED were still reported in China since late 2010. It resulted in high rates of morbidity and mortality in suckling piglets and substantial economic losses in the country. Both modified live vaccines and killed vaccines were commercially available in China, but the current vaccines were less effective against the PEDV variants, and the PED epidemics were not properly controlled. This may be attributed to antigenic and genetic differences between the vaccine strains and the epidemic strains [18,19,20]. Thus, the need for the development of better vaccines against the novel PEDV is urgent in the future.

Pro-inflammatory cytokines (IL-1β, IL-6, TNF-α) are a class of endogenous production produced by cells of the immune system that have many powerful biological effects that mediate a variety of immune responses, including controlling viruses and preventing the spread of viruses [21,22,23]. Type I interferon (IFN-α/β) is a group of structurally similar, functionally similar, low-molecular glycoproteins, which are the first lines of host defense against virus infection [24]. PEDV could inhibit type I interferon (IFN-α/β) and pro-inflammatory cytokines at the transcriptional level by blocking IκBα phosphorylation [25,26]. However, the ability of PEDV variant strains and successive passage strains to induce the production of type I interferon (IFN-α/β) and pro-inflammatory cytokines in host cells has not been reported.

In the present study, we isolated a virulent PEDV from clinical samples of diseased suckling piglets that showed severe vomiting, diarrhea, and dehydration. Analysis of the S gene indicated that the isolated virus SDSX16 belongs to the G2 genotype. Following over 100 successive passages on Vero cells, the adapted SDSX16 virus replicated to high titers compared with wildtype virus. We further uncovered that there were continuous 18 nucleotides (nt) deletion in the spike gene, which caused a six amino acid deletion in the SDSX16-P64 and significantly higher titer than that SDSX16-P63. Compared with SDSX16-P10, the pathogenicity of SDSX16-P75 was weakened, and porcine intestinal epithelial cells, infected with SDSX16-P75, produced higher levels of cytokines, not only at the mRNA levels, but also at the protein levels. Our research found that the deletion of 6 amino acids in the S gene may be associated with increased viral replication in cells rather than attenuation in pigs. These data will lay the foundation for future research on targeted PEDV effective vaccines.

## 2. Materials and Methods

### 2.1. Cells and Antibody

Vero cells (ATCC CCL-81) were stored in the Shandong Provincial Key Laboratory of Animal Diseases Control and Breeding, and Porcine intestinal epithelial cells were obtained from China General Microbiological Culture Collection Center (CGMCC NO. 11496). These cells were cultured using Dulbecco’s modified Eagle’s medium (DMEM, Gibco, Waltham, MA, USA) supplemented with 10% fetal bovine serum (FBS, Gibco, Waltham, MA, USA) and 1% penicillin/streptomycin in a humidified 5% CO_2_ incubator at 37 °C. Anti-PEDV spike-specific monoclonal antibody (MAb) was obtained from Mendian Diagnostics (Cat#9191, 3F12, Korea).

### 2.2. Clinical Samples Collection

Intestine samples and faeces were collected from piglets with diarrhea in a pig farm during a large-scale outbreak in Shandong province, China in 2016. The samples were PEDV-positive, but negative to transmissible gastroenteritis virus (TGEV), porcine rotavirus (PoRV), and porcine delta virus (PDCoV) was by using multi-RT-PCR. Small-intestine tissues were used to generate a 10% (*w*/*v*) homogenate in Earle’s balanced salt solution (Sigma-Aldrich, St. Louis, MO, USA). The suspension was centrifuged at 4000× *g* for 10 min at 4 °C. The supernatant was filtered through a 0.22 µm-pore-size filter and used for virus isolation. A one-tenth gram of feces was suspended in 1 mL phosphate-buffered saline (PBS), vortexed for 5 min, and then centrifuged at 4000× *g* for 5 min. The supernatant was filtered through a 0.22 µm-pore size filter and used as an inoculum for virus isolation.

### 2.3. Virus Isolation and Propagation

The virus was isolated in Vero cells (ATCC CCL-81), as described previously with some modifications [27,28]. Vero cells were seeded at 70% confluency in 6-well plates (Corning, NY, USA) and incubated overnight. After washing three times with PBS (pH = 7.4), cells were inoculated with 400 µL inoculum diluted in 200 µL of maintenance medium with trypsin (MMT) containing DMEM, 0.3% tryptose phosphate broth (TPB, Sigma-Aldrich, St. Louis, MO, USA), 3 µg/mL of trypsin and 1% penicillin/streptomycin, were added to each well [7,28,29]. After incubation 2 h at 37 °C, 5% CO_2_, the inoculated cells were maintained at 37 °C under 5% CO_2_ and monitored daily for cytopathic effects (CPE). When 80% of CPE appeared, cells were subjected to three rounds of freeze and thaw. The culture supernatants were then collected and centrifuged at 500× *g* for 10 min. The supernatants were aliquoted and stored at −80 °C until use. This was designated as ‘passage 1 (P1)’ viral stock, then confluent Vero E6 cells in T25 flasks were infected by PEDV strain at an MOI of 0.01. When extensive CPE was observed, the cell culture supernatant was harvested and used for the next passage in Vero E6 cells.

### 2.4. Growth Kinetics and Virus Titration

To conduct growth kinetics experiments, Vero cells cultured in 6-well-plates were infected with the SDSX16 isolate from different passages at an MOI of 0.01 and incubated at 37 °C under 5% CO_2_. The culture supernatants were harvested from at different time points (6, 12, 18, 24 and 48 h) and stored at −80 °C. Virus titers were measured in 96-well plates by 10-fold serial dilutions of samples, in triplicate per dilution. The Vero E6 cells were seeded in a 96-well plate at a density of 1 × 10^5^ cells/well in 200 µL and incubated for 24 h at 37 °C in 5% CO_2_. Ten-fold serial dilutions of the virus were added to the prepared wells with 100 µL of diluted virus per well. The tissue culture with 50% infective dose (TCID_50_) was expressed as the reciprocal of the highest dilution showing CPE by the Reed–Muench method [30].

### 2.5. Immunofluorescence Assay (IFA)

The Vero cells infected by PEDV (MOI = 0.01) in 24-well plates for 48 h were fixed with 4% paraformaldehyde for 15 min at room temperature (RT). Cells were permeabilized with 0.5% Triton X-100 in PBS at 4 °C for 15 min and were blocked with 1% bovine serum albumin (BSA) in PBS for 30 min at RT. The monoclonal antibody specific against PEDV spike protein was added to the cells as primary antibodies and incubated for 1 h at 37 °C. After washing three times in PBS, cells were incubated for 1 h at RT with dilution 1:1000 of the FITC-conjugated donkey anti-mouse IgG antibody (ThermoFisher, Waltham, MA, USA), followed by counter-staining with 4, 6-diamidino-2-phenylindole (DAPI, Sigma-Aldrich, St. Louis, MO, USA). The coverslips were mounted on microscope glass slides in mounting buffer and staining was visualized using a fluorescence Leica DM IL LED microscope (Leica, Wetzlar, Germany). ImageJ software version 1.51p was used to calculate the percentage of CPE cells per 200 cells in the visual field.

### 2.6. Morphologic Observation of Virus by TEM

Vero cells were seeded in 6-well plates and incubated overnight until the cell density reached 80% confluency. After washing with PBS, cells were inoculated with SDSX16-P5 at a multiplicity of infection (MOI = 1) diluted in MMT. After 2 h of incubation at 37 °C, cells were washed with PBS and then cultured with MMT. Cells were observed daily to check for any cytopathic effect (CPE), particularly syncytium formation. When CPE was obvious, the supernatant was discarded and the cells were collected by gently blowing with a cell scraper. The cells were collected in sterile tubes by centrifugation at 3000× *g* for 10 min and then supernatants were discarded. Cell pellets were washed with PBS and fresh 3% glutaraldehyde fixative was added and incubated for 3 h at 4 °C. The conventional TEM sample preparation method was followed for rinsing, 1% osmium acid (OsO4) fixed, drift-washed, dehydrated, soaked, Epon 812 embedded, semi-thin sliced with LKB-V-type ultra-thin Slicer line ultra-thin slices, lead citrate and uranium acetate dyes, and examined by JEOL-1200EX electron microscopy. Images were recorded using MORADA-G2.

### 2.7. RNA Extraction, RT-PCR and Sequence Analysis

Virus RNA was extracted from the supernatants of homogenized samples using Trizol reagent according to the manufacturer protocols (ThermoFisher, Waltham, MA, USA). Complementary DNA (cDNA) was produced through reverse transcription, using PrimeScript™ One-Step RT-PCR Kit Ver.2 according to the manufacturing protocols (TaKaRa Bio, Otsu, Shiga, Japan). Targeted sequence primers of the PEDV S gene and the whole gene were amplified by PCR, with reference to the PEDV CV777 strain (GenBank: AF353511.1). The S1 fragment was amplified using primers PEDV-S1F: 5′-GTGGCTTTTCTAATCATTTGGTC-3′ and PEDV-S1R: 5′-CTGGGTGAGTAATTGTTTACA-3′, under the following conditions: reverse transcription at 50 °C for 30 min, denaturation at 95 °C for 2 min, 35 cycles of 94 °C for 30 s, 50 °C for 30 s, and 72 °C for 3 min, and 72 °C for 10 min. The product fragment was 2508 bp [31]. The S2 fragment was amplified using primers PEDV-S2F: 5′-GGCCAAGTCAAGATTGCACC-3′ and PEDV-S2R: 5′-AGCTCCAACTCTTGGACAGC-3′, under the following conditions: reverse transcription at 50 °C for 30 min, denaturation at 95 °C for 2 min, 35 cycles of 94 °C for 30 s, 52 °C for 30 s, and 72 °C for 2 min, and 72 °C for 10 min. The product fragment was 1850 bp. The whole genome of PEDV was amplified using primers in Table 1, the reaction conditions are as follows: Reverse transcription at 50 °C for 30 min, denaturation at 95 °C for 2 min, 35 cycles of 94 °C for 30 s, 52 °C for 30 s, and 72 °C for 1 min 30 s, and 72 °C for 10 min.

The RT-PCR products were identified by electrophoresis on a 1% agarose gel, and cloned into the pMD19/T vector (TaKaRa Bio, Otsu, Shiga, Japan). The positive clones were sequenced at the Shanghai Sangon Biological Engineering Technology & Services Co., Ltd. (China). Sequence assembly was carried out using Lasergene Software version 7.1. Moreover, sequence alignments and phylogenetic trees of the S genes and the whole SDSX16 genomes were also performed using distance-based neighbor-joining method of MEGA4.0 software. The numbers at each branch represent bootstrap values greater than 50% of 1000 replicates.

### 2.8. Pig Infection Experiment

The three-day-old Duroc crossbred piglets were obtained from the Shandong Academy of Agricultural Science (Jinan, Shandong, China). All piglets were confirmed to be free of PEDV, TGEV, PoRV, and PDCoV infections using a viral serum neutralizing test for the detection of antibodies against PEDV, TGEV, PoRV, and PDCoV, as well as RT-PCR to detect viral nucleic acids. To determine the virulence and pathogenesis of the PEDV isolate and successive passage strains, SDSX16 variants at five passage levels (SDSX16-P10, -P30, -P63, -P64, -P75) were selected for pig study. Thirty three-day-old Duroc crossbred piglets were randomly divided into six groups (P10, P30, P63, P64, P75, and Mock), each group contained five piglets. The infected group (P10, P30, P63, P64, and P75) were orally challenged with P10, P30, P63, P64, and P75, respectively (10^6^ TCID_50_/piglet), while the uninfected group (Mock) was orally given maintenance medium in the same route. These piglets were observed and recorded daily for signs of vomiting, diarrhea, and body weight until the end of the experiment and humanely euthanized following the appearance of severe diarrhea or after becoming moribund. At the end of the study, all surviving piglets were euthanized humanely.

At necropsy, intestinal tissue specimens (<0.3 cm thick) were collected from each piglet and were fixed with 10% formalin for 24 h at RT and embedded in paraffin according to the standard laboratory procedures. The formalin-fixed paraffin-embedded tissues were cut in 5 µm thick on a microtome (Leica), floated on a 40 °C water bath containing distilled water, and transferred onto glass slides. The tissues were then deparaffinized in xylene for 5 min and washed in decreasing concentrations of ethanol (100%, 95%, 90%, 80%, and 70%) for 3 min each. The fixed intestine sections were evaluated for PEDV antigen by immunohistochemistry (IHC) using 200× diluted PEDV-specific monoclonal antibodies (1:200) with the antigen retrieval method described previously [32]. This study was approved by the local animal ethics committee.

### 2.9. Detection of Cytokines

Porcine intestinal epithelial cells were cultured in 6-well plates at a concentration of 10^6^ cells per well, were infected with SDSX16-P10, -P63, -P64, -P75 (MOI = 1) respectively. The porcine intestinal epithelial cells in 6-well plates were transfected with poly(I:C) (5 µg/mL) (Sigma-Aldrich, St. Louis, MO, USA) were served as a positive control group by using Lipofectamine 3000 transfection reagent (ThermoFisher, Waltham, MA, USA). Hours post-infection, the cells were collected for real-time qPCR and Flow cytometry to detect cytokines. To detect mRNA levels of cytokines, total RNA was extracted with Trizol reagent according to the manufacturer protocols (ThermoFisher, Waltham, MA, USA) and subjected to reverse transcription with PrimeScript RT Master Mix (TaKaRa Bio, Otsu, Shiga, Japan). Measurements were performed using the SYBR Premix Ex Taq (TaKaRa Bio, Otsu, Shiga, Japan ) on a LightCycler 480 II with a 96-well plate block (Roche, Switzerland). Amplification was performed for 30 s at 95 °C, followed by 40 cycles of 95 °C for 5 s, 60 °C for 30 s. The relative expression of mRNA was quantified within each sample using the 2^−ΔΔ*C*t^ method, and the cut-off Ct value was 35. β-actin mRNA expression levels were used as a housekeeping gene for internal control. Primers for each cytokine and gene were listed in Table 2.

To further confirm the cytokine results, the infected porcine intestinal epithelial cells were subjected to flow cytometry. Briefly, the infected cells were fixed and permeabilized by BD Cytofix/CytopermTM (BD Biosciences, East Rutherford, NJ, USA). After twice wash with phosphate buffer solution, the fixed cells were stained with anti-TNF-α-FITC (Novus Biologicals, Littleton, CO, USA) or anti-IL-6-FITC (R&D Systems, Minneapolis, MN, USA) in the dark for 30 min at RT, then the cells samples were washed and resuspended with PBS, and analyzed on FACS AriaTM II (BD Biosciences, East Rutherford, NJ, USA) with the FlowJo software (version 10.0; Treestar, USA).

### 2.10. Statistical Analysis

Statistically significant differences were determined by Student’s t-test using GraphPad Prism software (version 8.0; San Diego, CA, USA). Two-way ANOVA was used to calculate statistical significance, and multiple samples were compared by one-way ANOVA. Values are expressing as mean ± SEM of at least independently repeated three times unless otherwise noted, and a *p*-value below 0.05 was considered statistically significant (*p < 0.05, **p < 0.01, ***p < 0.001).

## 3. Results

### 3.1. Virus Isolation and In Vitro Characterization

**Figure 1:**
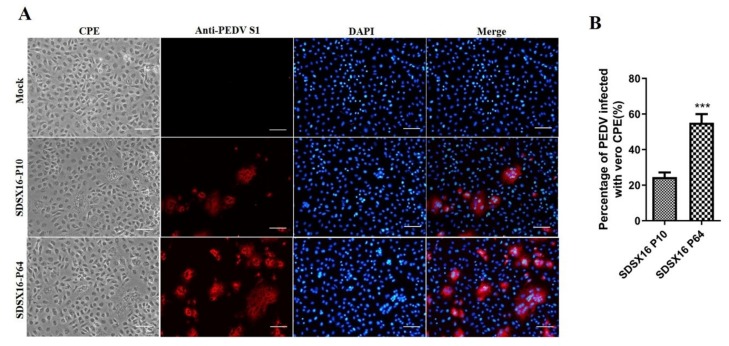
Cytopathology and immunofluorescence assay (IFA) of PEDV isolate SDSX16 in Vero cells. (**A**) Vero cells were infected with PEDV SDSX16-P10 and SDSX16-P64 isolates. PEDV-specific CPEs were monitored daily and were photographed at 48 h post-infection (hpi) using an inverted microscope (first column). For immunostaining, infected cells were fixed at 48 hpi and incubated with MAb against the S protein, followed by FITC conjugated donkey anti-mouse IgG antibody (second column). The nuclei were counterstained with DAPI (third column) and examined using a fluorescence microscope. Scale bar represents 20 µm. (**B**) Statistical results of the percentage of total cell number of cells producing CPE infected by PEDV in the visual field. Data are shown as mean ± SEM (***p < 0.001).

We tried to isolate PEDV from RT-PCR-positive clinical samples in Vero cells. One PEDV isolate designated as SDSX16 was successfully isolated from the feces of a naturally infected piglet. This sample was originated from a commercial swine farm located in Shandong Province and was obtained on September 16, 2016. This SDSX16 virus and it could produce distinct CPEs from passages 5 (P5), typical of PEDV infection, such as cell fusion, syncytia formation, and detachment in infected Vero cells. We then investigated whether the isolate could be efficiently cultivated and maintained in cultured cells. To do this, the isolated PEDV strain SDSX16 was serially passaged in Vero cells for a total of 100 passages (P1 to P100). The time for the onset of CPE was typically 24 h and prominent CPE was observed within 48 h post-inoculation. The development of CPE was faster in later passages. The visible CPE appeared at 18 h and became predominant by 36 h. Virus propagation was confirmed by detecting PEDV antigens by IFA using an anti-PEDV spike-specific monoclonal antibody. The distinct staining pattern for typical syncytia formation was distributed in the cytoplasm. In contrast, neither CPE nor spike-specific monoclonal antibody staining was detected in mock-inoculated cells. Examples of CPE, and corresponding IFA images in cells inoculated with passages SDSX16-P10 and -P64, are shown in Figure 1.

**Figure 2:**
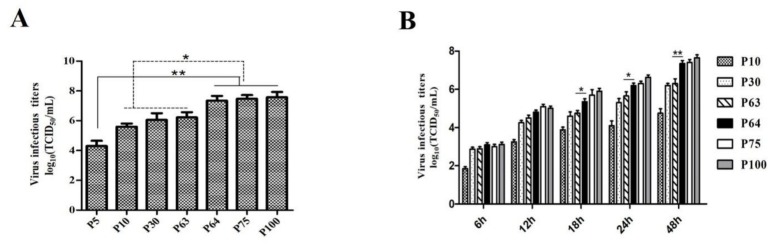
Growth kinetics of SDSX16 in Vero cells. (**A**) Virus titers of different passages. Vero cells were infected with PEDV SDSX16 and harvested at indicated passage numbers. At 48 h of infection for each passage, supernatants were collected and virus titers were determined. Data are shown as mean ± SEM (* *p* < 0.05, ** *p* < 0.01) (**B**) One-step growth for SDSX16. Vero cells were infected with PEDV SDSX16 (P10, P30, P63, P64, P75, P100), and at the indicated time points, post-infection, culture supernatants were harvested and virus titers were determined. The 50% tissue culture infectious dose (TCID_50_) was calculated. Results are expressed as the mean values from triplicate wells. Data are shown as mean ± SEM (* *p* < 0.05, ** *p* < 0.01).

The titer of the SDSX16 was measured every ten generations. The virus titers ranged from 10^4.15^ to 10^4.50^ TCID_50_/mL up to SDSX16-P5, and the peak viral titer reached 10^7.6^ TCID_50_/mL after passage 100. The titer of SDSX16-P70 (7.44 log_10_TCID_50_/mL) was approximately 10-fold higher than that of SDSX16-P60 (6.22 log_10_TCID_50_/mL). By retrospective testing, we found that the titer of SDSX16-P64 (7.22 log_10_TCID_50_/mL) was significantly higher than that of SDSX16-P63 (Figure 2A). The growth kinetics study further showed that SDSX16 replicated rapidly and efficiently in Vero cells, reaching a maximum titer >10^7.0^ TCID_50_/mL by 48 h of inoculation, and the titer of SDSX16-P64 began to be significantly higher than the titer of SDSX16-P63 by 18 h of inoculation (Figure 2B).

### 3.2. Morphologic Characterization of Virus

**Figure 3:**
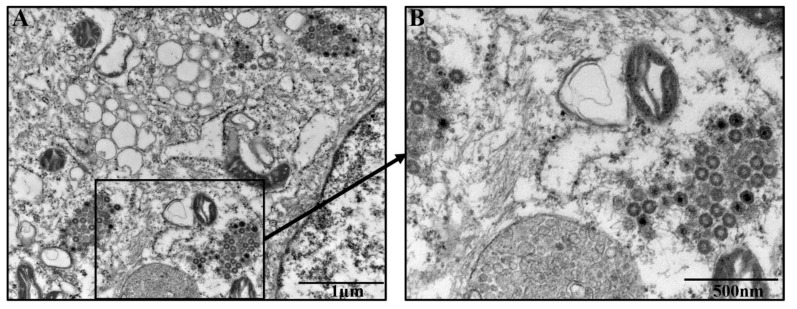
Electron micrographs of PEDV SDSX16-P5-infected Vero cells. (**A**) Virus-particles were found in the cytoplasm of Vero cells. (**B**) Enlarged view of the boxed area in (**A**). Black arrow indicates the presence of PEDV particles. Bars represent 1 µm (**A**), and 500 nm (**B**), respectively.

The morphology of PEDV SDSX16-P5 was characterized in Vero cells by electron microscopy. The virus-induced intracellular membrane structures were captured with a bright domain in the cytoplasm. The presence of clustered spherical particles in loose membrane vesicles was found in the cytoplasm of Vero cells infected by PEDV SDSX16-P5, showing a typical shape of coronaviruses (Figure 3).

### 3.3. Sequence Analysis

**Figure 4:**
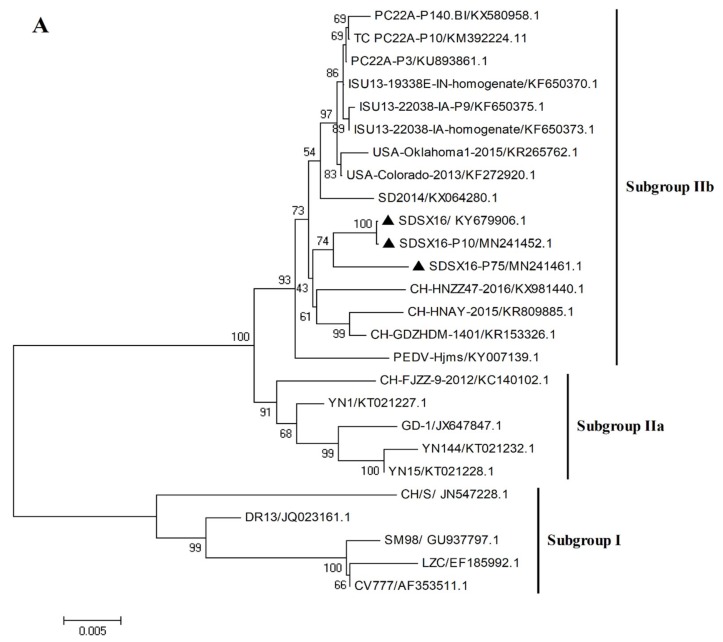

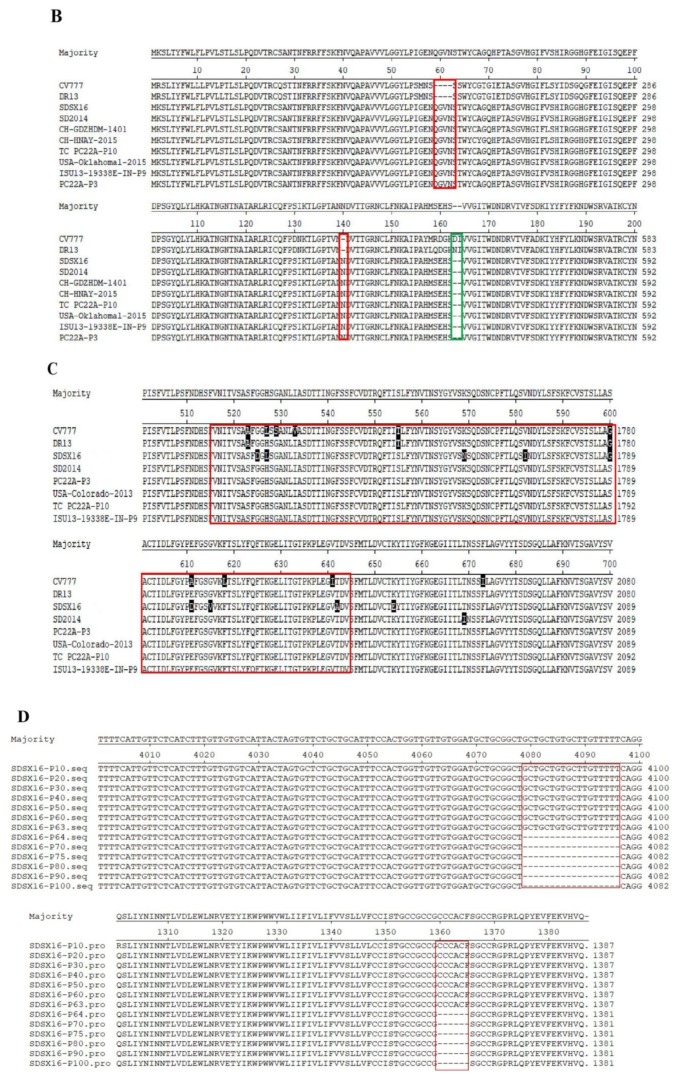
Phylogenetic analysis of S genes of the PEDV SDSX16 (▲). (**A**) Phylogenetic trees generated by the neighbor-joining method with nucleotide sequences of the full-length S gene. Bootstrapping with 1000 replicates was performed. (**B**) PEDV SDSX16 S1 domain and neutralizing epitopes (COE-Black). The red and green boxes represent the insertion and deletion of nucleotides, respectively. (**C**) were analyzed by the MegAlign software of Lasergene 7.1. The red boxes represent the core of epitope (COE). (**D**) Alignments of the S proteins nucleotides and amino acid sequences of SDSX16-P10, -P20, -P30, -P40, -P50, -P60, -P63, -P64, -P70, -P80, -P90, -P100. The red boxes represent the nucleotides deletion.

Sequencing of the spike gene of the SDSX16 isolate (GenBank number: KY679906.1) yielded a sequence of 4161 bp, which was analyzed through a comparison with 23 typical strains available in NCBI. The phylogenetic tree (Figure 4A) revealed that these PEDV strains could be divided into Subgroup I and Subgroup II. Amino acid analysis indicated that the SDSX16 isolate shared 93.5%~94.9%, 97.5%~98.4%, and 98.5%~99% identity to Subgroup I, Subgroup IIa, and Subgroup IIb, respectively. The major variable region of the S gene was the S1 domain. Compared with Subgroup I (CV777 and DR13) and Subgroup IIb (SDSX16, SD2014, and PC22A-P10 et al.) [14,18,19,33] isolates exhibited an insertion of five amino acids at 59–62 position, 140 position, as well as the deletion of two amino acid at 163–164 position (Figure 4B). The same insertions and deletions have been found in other variant strains isolated, indicating that epidemics are caused by variant strains in many countries. Moreover, in comparison with the CV777 strain, some amino acid substitutions in the neutralizing region of the core of epitope (COE) (499–638aa) were observed [34,35] and the regions of the aligned sequences, corresponding to these regions were COE (514–642aa, Figure 4C). The sequence analysis revealed that the other variant strains had the same COE mutations. The S gene of SDSX16 at different passage levels (SDSX16 P10-P100 GenBank number: MN241452-MN241464) were analyzed. The results showed that the spike gene had an 18 nt deletion resulting in the deletion of 6 amino acids when the SDSX16 was passaged to the P64, and this deletion was stable until the P100 (Figure 4D).

The complete gene of SDSX16 was analyzed through a comparison with 6 typical strains available in NCBI (CV777, GenBank number: AF353511.1, CH/S, GenBank number: JN547228.1, SM98, GenBank number: GU937797.1, LZC, GenBank number: EF185992.1, USA/Colorado/2013, GenBank number: KF272920.1, SD2014, GenBank number: KX064280.1). The nucleotide identity of SDSX16 and CV777, CH/S, SM98, LZC, USA/Colorado/2013 and SD2014 were 96.7%, 96.4%, 97.2%, 96.8%, 99.2%, and 99.6%, respectively. Compared with the complete gene of SDSX16-P10, the amino acid analysis exhibited ORF1a and ORF1b of SDSX16-P64 have five-point mutants (L449I, K907R, I1466T, S2052C, M3034S); the S gene mainly has ten-point mutations (Q70R, D139N, L269V, K354E, M487I, V707A, Q825H, T921S, I1063F, L1347F) and six amino acid deletions (1360CCCACF1365); the M, and E genes have one-point mutant (S207A) and (E62F), respectively; the N gene has three-point mutants (N123T, D400E, S412V); the ORF3 gene has eight-point mutants (L25S, I70V, V80F, C107F, L148E, F159S, D168N, Q182H) in Supplementary Table S1. Interestingly, the amino acid sequence analysis results of SDSX16-P63, -P64, -P75 showed no mutations in ORF1a/b, M, N, E, and ORF3 genes. Also, the S gene of SDSX16-P64 mainly lost six amino acids than the -P63 S gene and the S gene of -P75 had no amino acid deletion or addition compared to the S gene of -P64. These results suggest that the biggest difference in the genomes of SDSX16-P64 and -P63 is the 6 amino acid deletion in -P64.

### 3.4. Pathogenicity of SDSX16

**Figure 5:**
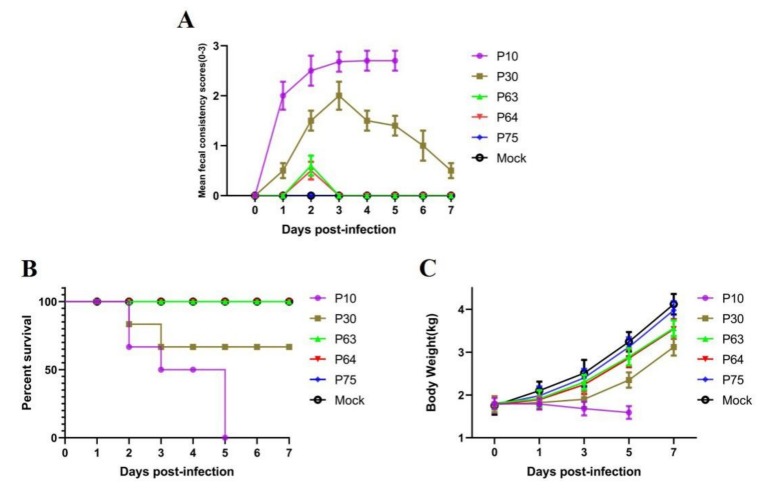
Pathogenicity analysis of SDSX16 different generation strains. (**A**) The clinical average score of diarrheas in different groups (SDSX16-P10, -P63, -P64, -P75, and Mock). The severity of diarrhea was scored based on visual examination; 0 = no diarrhea; 1 = mild diarrhea; 2 = fluidic diarrhea; 3 = severe watery diarrhea. (**B**) Piglets infected with SDSX16-P10, -P63, -P64, -P75 survival was monitored. Five piglets per group. (**C**) The average body weight changes in each group.

To determine the virulence and pathogenesis of the PEDV isolate and different passage strains, we have conducted oral inoculation of nursery pigs with PEDV SDSX16-P10, -P30, -P63, -P64, -P75, respectively. We found that piglets (group A) orally challenged with of SDSX16-P10 (10^6^ TCID_50_/piglet), developed diarrhea at 18 h, furthermore, the infected piglets presented with anorexia and depression, and the piglets began to exhibit watery diarrhea at 2 days post-challenge (Figure 5A). Two piglets died at 2 days post-challenge and all piglets died at 5 days post-challenge in group A (Figure 5B). Whereas, the piglets (group B) started to vomit 24 h after orally inoculated with the -P30 and showed diarrhea at 30 h and some animal’s clinical signs of vomiting after suckling. However, there was no significant difference in the clinical examination between groups C and D, infected by -P63, and -P64, respectively, and piglets were found to have slight diarrhea at 48 h. The piglets’ group E challenged with P75 virus was not showing any significant diarrhea symptom. No, any clinical signs were observed in group F. In this animal experiment study, there was a significant decrease in weight post-challenge in the P10, P30 infected group, whereas the uninfected piglets gained weight (Figure 5C). No obvious temperature changes were observed between these groups.

**Figure 6:**
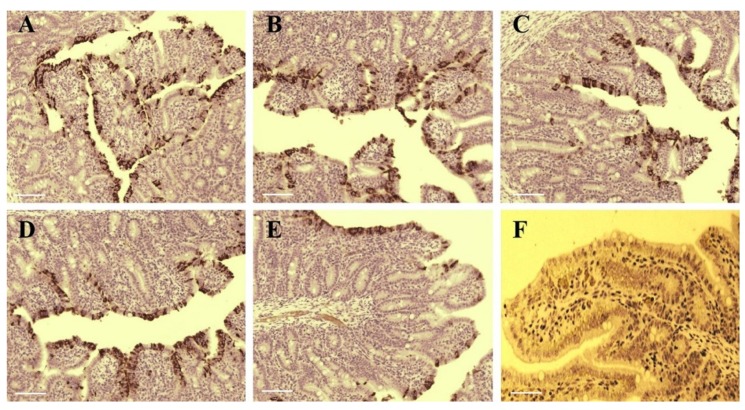
The tissues of infected PEDV SDSX16 isolate strain and uninfected groups were intestine histologically examined. (**A**–**E**) were infected withSDSX16-P10, -P30, -P63, -P64, and -P75, respectively. The immune-histochemical method was used to detect positive infection cells present signal in a segment of the intestines. (**F**) was the uninfected group from control animals, and there was no significant change. Scale bar represents 50 µm.

Immunohistochemistry of PEDV confirmed the presence of the virus in the cytoplasm of epithelial cells on atrophic villi in some segments of the small intestine (Figure 6A–E). PEDV antigen was detected in some cells of ileum, jejunum, duodenum, and cecum of piglets infected by SDSX16-P10, P30, P64, P65 or P75. SDSX16-P10 and -P30 caused severe villous atrophy in the cytoplasm of villous enterocytes in most jejunum and ileum. P64 and P65 caused moderate villous atrophy in some jejunum and ileum. P75 caused mild villous atrophy in a few jejunum or ileum. PEDV IHC antigen was not detected in the intestines of mock piglets (Figure 6F). The piglets infected with SDSX16-10 developed severe acute enteritis typical of PEDV throughout the study, demonstrating that the isolate was virulent and enteropathogenic in neonatal piglets. The piglets were infected by the higher passage of SDSX16, which had slighter diarrhea, vomiting and fecal shedding. The results indicated that the mutations in the S gene, caused by the serial passages of SDSX16, might be related to the attenuation of virulence in vivo.

### 3.5. Analysis of Cytokine Expression by SDSX16 Infected Porcine Intestinal Epithelial Cells

**Figure 7:**
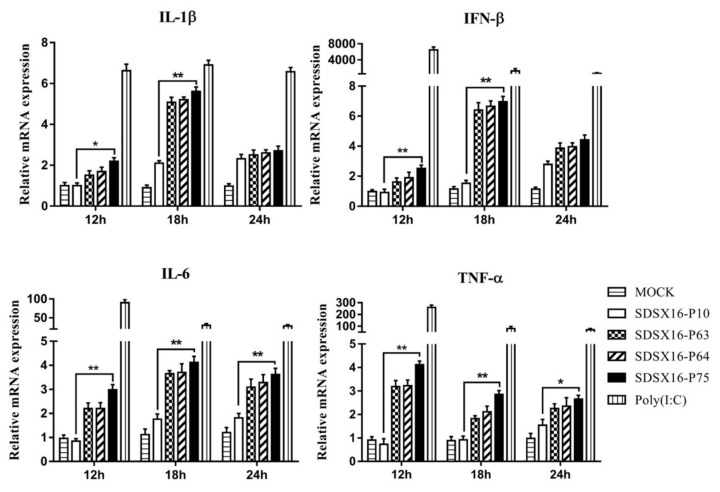
Expression of type I interferon and proinflammatory cytokines mRNA levels in porcine intestinal epithelial cells induced by different passage SDSX16 strains. The cells infected with PEDV SDSX16-P10, -P63, -P64, -P75 at an MOI of 1, MMT (Mock) or transfected with poly(I:C) were collected at 12, 18, 24 h post-infection (hpi). Relative IL1β, IL6, TNF-α, and IFN-β mRNA expression was determined by qPCR. The data are shown as fold changes and were normalized to control cell data. The significant difference between PEDV SDSX16-P10 and -P75 was expressed with their *p* values. The mean ± SEM of data from three independent experiments are shown. (* *p* < 0.05, ** *p* < 0.01).

Pro-inflammatory cytokines and type I interferons are produced at the porcine intestinal epithelial cells as part of the innate immune response during the infection process. We evaluated the changes in gene expression of pro-inflammatory cytokines (IL1β, IL6, and TNF-α) and type I interferon (IFN-β) in porcine intestinal epithelial cells infected with different SDSX16 strains. The mRNA levels of IL1β, IL6, TNF-α and IFN-β in porcine intestinal epithelial cells infected with SDSX16-P10 strain were much lower than SDSX16-P75 at 18 h post-infection (hpi). However, there was no significant difference in the examination of cytokine expression between porcine intestinal epithelial cells infected by P63 and P64 respectively (Figure 7).

**Figure 8:**
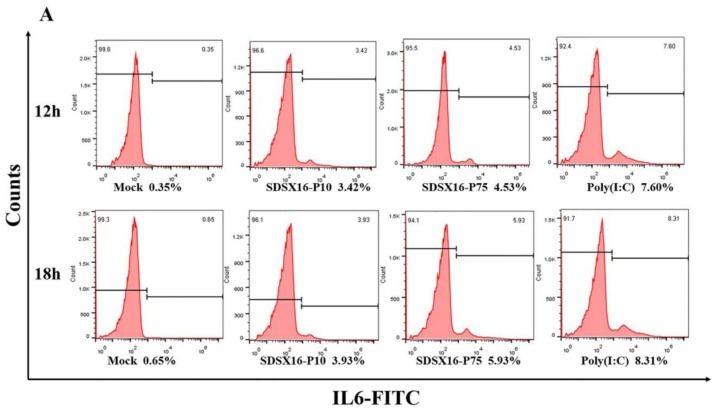

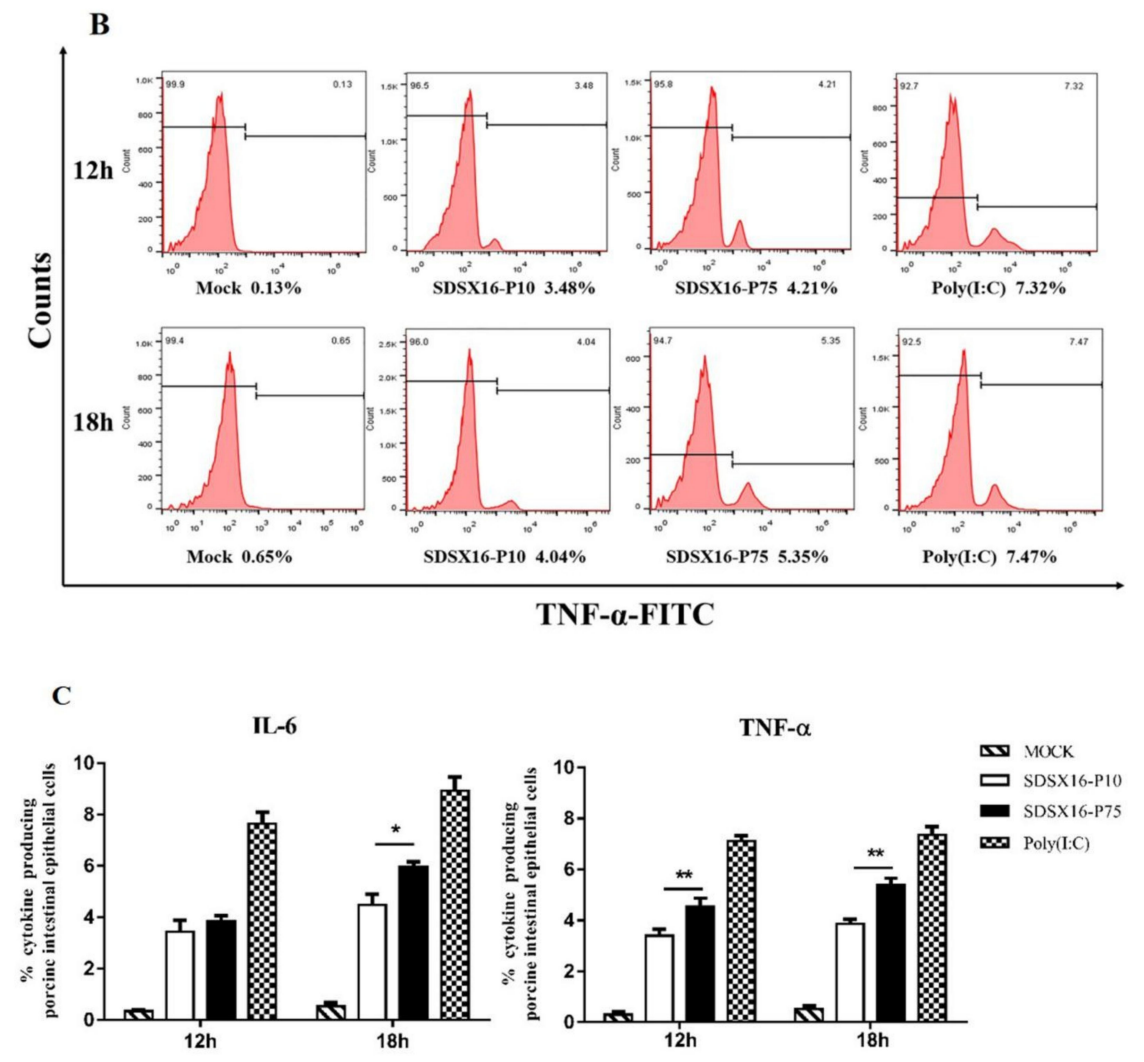
IL6 and TNF-α protein levels on porcine intestinal epithelial cells infected with SDSX16-P10 or -P75. The cells infected with PEDV SDSX16-P10, -P75, MMT or transfected with poly(I:C) were collected at 12 and 18 hpi. IL6 and TNF-α protein levels on porcine intestinal epithelial cells were determined individually by flow cytometry. (**A**,**B**) One representative experiment was shown. (**C**) The data indicated the mean  ±  SEM of three independent experiments, * *p* < 0.05; ** *p* < 0.01.

Next, we used flow cytometry to detect IL6 and TNF-α protein levels in cells infected with SDSX16-P10 or -P75. The results showed that SDSX16-P10 strain-stimulated IL6 and TNF-α expression of the protein was markedly lower than SDSX16-P75 at 18 hpi (Figure 8). Collectively, these data suggest that there was no significant difference in cytokines expression between cells infected with SDSX16-P63 and -P64, and SDSX16-P10 had a stronger ability to inhibit cytokines expression than SDSX16-P75.

## 4. Discussion

The PEDV variant strains have been detected in many provinces in China and have become one of the most economically important viral causes for diarrhea in piglets [36,37]. At present, piglet diarrhea, caused by PEDV infection, leads to huge losses in many pig farms [38]. We collected clinical samples from pig breeding farms in Shandong Province where severe acute diarrhea was associated with high mortality in suckling piglets. Isolation of variant strains is important for effective control of piglet PEDV infection and vaccine development. In this study, we successfully isolated a strain of PEDV from the intestinal tissues of a PED-infected piglet. The PEDV SDSX16 isolate showed obvious CPE (syncytium formation) in Vero cells from after passage 5, the viral titers started from roughly 10^4.15^ TCID_50_/mL and increased after serial passages to 10^7.6^ TCID_50_/mL. Phylogenetic analysis based on the S gene sequences showed the SDSX16 isolate belonged to subgroup II. More recently, it has been reported that PEDV G2 strain-based inactivated vaccine candidates are candidates for developing an effective vaccine against the virulent pandemic PEDV strains [39]. These results suggest the SDSX16 strain could be a candidate strain for vaccine development against PEDV variant strains.

To develop the PEDV vaccine, we attenuate the virulence of the SDSX16 strain by the successive passage in vitro. Considering that S protein plays a crucial role in mediating virus-cell fusion and inducing neutralizing antibodies, we focus on analyzing to vary the S gene during the successive passage. The result found the S gene had greater diversity, while other genes (ORF1a/b, M, N, E, and ORF3) occurred acid point mutants. We further determined the serial passage of SDX16 in Vero cells resulted in the viral growth adaptation and promoting its replication in vitro by growth kinetics and virus titration assay. The serial passage of PEDV and other coronaviruses often results in the attenuation of virus virulence [40,41,42,43,44]. To study whether the serial passages of SDSX16 have influenced the viral pathogenicity, PEDV-seronegative, three-day-old pigs were inoculated orally with SDSX16 different viruses. The results suggested that successive passage resulted in attenuation of the SDSX16 strain, and deleting six amino acids in the S gene might affect the pathogenicity of the virus. Although data indicating pivotal roles of S protein in determining phenotypes of coronaviruses have been accumulated, it remains to be determined whether the mutations in the S gene of SDSX16 contribute to the viral attenuation and virus virulence. Therefore, we will need to apply PEDV reverse genetics systems to further studies to define the genetic mechanisms that underlie the attenuation of PEDV.

It has been widely reported that type I interferons (IFN-α/β) are two essential cytokines that can control viral infection [45]. TNF-α has an antiviral function, and IL6 is associated with improving humoral and mucosal immune response [21,46]. Variant PEDV (genogroup G2) exhibits higher resistance to the interferon than the existing PEDV and avoids the neutralizing antibody against PEDV [47,48,49]. In order to understand whether there were differences in the cytokine levels between the SDSX16-P10 and -P75 strain. We compared SDSX16-P10 with -P75 and found that IFN-β mRNA levels could be up-regulated in porcine intestinal epithelial cells. However, the mRNA and protein levels of pro-inflammatory cytokines, in cells induced by SDSX16-P75, were much higher than SDSX16-P10. Additionally, it has been reported that highly pathogenic PEDV strain suppressed the induction of pro-inflammatory cytokines and type 1 interferon production through the down-regulation of Toll-like receptors (TLRs) [25,26,50,51,52]. SDSX16-P75 induces higher levels of cytokine production indicating that the pathogenicity of the SDSX16 strain is weakened by the successive passage in vitro, which is consistent with the results of animal experiments. Our study demonstrated that the ability of PEDV to induce type I IFNs is strain-specific. Thus, Type I IFNs may play an important role in PEDV replication and pathogenesis.

In summary, this study explored the virulent PEDV SDSX16 strain that was isolated in Shandong Province, China. The mutants of the S gene maybe improve the virus replication capability in Vero cells it was passaged to the 64th generation and was not related to the virus virulence. SDSX16-P75 induced higher cytokines levels in porcine intestinal epithelial cells and highly attenuated compared to -P10. SDSX16-P75 may be a suitable candidate virus to develop a variant strain-based PEDV vaccine to control current PEDV outbreaks.

## Figures and Tables

**Table 1:** Primers for SDSX16 complete genes amplification.

Primer Name	Sequence 5′–3′	Size (bp)
PEDV-F1	CGCAAATGGGCGGTAGGCGTG	1169
PEDV-R1	GCATGAAGCCACAAGGAC	
PEDV-F2	GTTGTCTTGTCTGAGCCA	1119
PEDV-R2	CAGGATGTTCAATGACAA	
PEDV-F3	CAATGTGAATCTCGTCGT	1259
PEDV-R3	GATCCTGTGAGATCGTGTC	
PEDV-F4	CTCCCTTGAATTTGAGTTCG	1326
PEDV-R4	TGACACCACGTCCAACTTTA	
PEDV-F5	CAGACGTTGAGCCTGTATT	1157
PEDV-R5	GTTGTCTGTGACAACGGT	
PEDV-F6	ATGTGGAGCGTTTCTACGCA	1284
PEDV-R6	AACATCATCACAAACAGGGC	
PEDV-F7	GCCTTTCGTAAGAGGGAT	1460
PEDV-R7	AGGGCAATGAAATCACGTA	
PEDV-F8	TTGCTGAGGCTCATCGTTA	1493
PEDV-R8	AGAGCCTACGAACTTGTCA	
PEDV-F9	TCGGTGATATGTCTGTTGGC	1346
PEDV-R9	TGCAGAGACTGGATTGAGGC	
PEDV-F10	TGGTACCGTTGAGTTTTGCT	1330
PEDV-R10	TTCATGCTAGAGAGACAGCC	
PEDV-F11	GCTTCTACGGTATTCTCTACT	1249
PEDV-R11	TGCACCATTAGGAGAATCCA	
PEDV-F12	CCTGGTAAGCTGAAGCAGCG	1462
PEDV-R12	AAGCACTCACTAGCAAGGCA	
PEDV-F13	ATTCATCGTGTCTATGCATTG	1367
PEDV-R13	TGCTCTGTCGCACTTTGGG	
PEDV-F14	CCATGACTACTCGGCAGTAT	1232
PEDV-R14	CACACATATGGAGTGATGGC	
PEDV-F15	AATCTGCAGGGCTTTGTGTT	1318
PEDV-R15	GACAGCGATAACACTTGTGC	
PEDV-F16	TGGTGGATGAGGTCTCTATG	1342
PEDV-R16	GGGCATGTTTGAAACAACAG	
PEDV-F17	GGTTGCGTTGTAACTGAGTC	1358
PEDV-R17	GTCCTACCTTACGTTTGGCA	
PEDV-F18	TCGGCGGGCTTTACTATTTG	1306
PEDV-R18	CACCAAGATGTAGCACACG	
PEDV-F19	CTCCAGACATTTTATCCGCA	1294
PEDV-R19	CGCGCAGTAGCATTAGTGTT	
PEDV-F20	GGCGGTTATCTACCTATTGG	1345
PEDV-R20	CTGTTCATGACTCAGAAGG	
PEDV-F21	TGTTGGACGCTGTCACAAT	1518
PEDV-R21	GACCATTAGAACAGCGCTTA	
PEDV-F22	TCACCCAGTACACTGCAGC	1412
PEDV-R22	GAGAGACTCTGAACGCTGC	
PEDV-F23	GCCTTGACTCTACGTGAGCC	1423
PEDV-R23	CGCTATTACACAACCGGTGA	
PEDV-F24	GACACTTTCTTTCCTCAATG	1475
PEDV-R24	AGTCCTATAGCGGAGGTCGG	
PEDV-F25	GGACGTGTTGGTCGTTCAGT	1242
PEDV-R25	CATTTGGATCAGACTTTGGC	
PEDV-F26	AGATGCGGAATTTGTCGAA	1121
PEDV-R26	GACTTCAAGTCACGTGAA	
5′UTR1	GCATCGCTCCATTATCC	623
5′UTR2	TTACCACCACGACGACC	
3′UTR1	GGCATAAGCAACAGCAG	690
3′UTR2	ATCGCCAGTTTAGCACCA	

**Table 2:** Primers used for Real-time PCR.

Gene	Sequence 5′–3′	GenBank NO.
IL-1β-F	GAAAGCCATACCCAGAGGTC	NM_214055.1
IL-1β-R	GCACTAATCTAGGGAAGACAGC	
IL-6-F	TGGGTTCAATCAGGAGACCT	AF518322.1
IL-6-R	CAGCCTCGACATTTCCCTTA	
TNF-α-F	CCCCCAGAAGGAAGAGTTTC	JF831365.1
TNF-α-R	CGGGCTTATCTGAGGTTTGA	
IFN-β-F	CTCCAAATCGCTCTCCTGAT	GQ415073.1
IFN-β-R	GGGACCTCAAAGTTCATCCT	
β-actin-F	TCTGGCACCACACCTTCT	AY550069.1
β-actin-R	GATCTGGGTCATCTTCTCAC	

## Supplementary Materials

Supplementary materials can be found at https://www.mdpi.com/1999-4915/12/4/391/s1. Fasta file: Fasta file for multipaired sequences using MAGE 4.0 software. Table S1: Summary of Signature Amino Acid Mutation in SDSX16-P10, SDSX16-P63, SDSX16-P64 and SDSX16-P75 Viruses.
