# Supplementary material for: Successive Passage In Vitro Led to Lower Virulence and Higher Titer of A Variant Porcine Epidemic Diarrhea Virus
Source: Viruses. 2020 Apr 1;12(4):391. doi: 10.3390/v12040391 (PMC7232491; doi:10.3390/v12040391)
Supplement: Supplementary file 1 [file viruses-12-00391-s001.zip › Supplementary Materials Table S1..pdf]

Table S1. Summary of Signature Amino Acid Mutation in SDSX16 Viruses

Gene	Amino Acid position	Virus			
		SDSX16-P10	GSDSX16-P63	SDSX16-P64	SDSX16-P75
ORF1a/b	449	L	I	I	I
	907	K	R	R	R
	1446	I	T	T	T
	2052	S	C	C	C
	3034	M	S	S	S
S	70	Q	R	R	R
	139	D	N	N	N
	269	V	V	V	V
	354	K	E	E	E
	487	M	I	I	I
	707	V	A	A	A
	825	Q	Q	H	H
	921	T	S	S	S
	1063	I	F	F	F
	1347	L	F	F	F
	1360-1365	CCCACF	CCCACF	deletion	deletion
M	207	S	A	A	A
ORF3	25	L	S	S	S
	70	I	V	V	V
	80	V	F	F	F
	107	C	F	F	F
	148	L	E	E	E
	159	F	S	S	S
	168	D	N	N	N
	182	Q	N	N	N
N	123	N	T	T	T
	400	D	E	E	E
	412	S	V	V	V
E	62	E	F	F	F
